# Mobilisation of HLA‐F on the surface of bronchial epithelial cells and platelets in asthmatic patients

**DOI:** 10.1111/tan.14782

**Published:** 2022-08-30

**Authors:** Sabrina Fiouane, Mohamad Chebbo, Sophie Beley, Julien Paganini, Christophe Picard, Xavier‐Benoît D'Journo, Pascal‐Alexandre Thomas, Jacques Chiaroni, Pascal Chanez, Delphine Gras, Julie Di Cristofaro

**Affiliations:** ^1^ CNRS, EFS, ADES, UMR7268 Aix Marseille University Marseille France; ^2^ Etablissement Français du Sang PACA Corse Marseille France; ^3^ INSERM 1263, INRAE 1260, C2VN Aix Marseille University Marseille France; ^4^ Xegen Gemenos France; ^5^ Department of Thoracic Surgery, North Hospital Aix‐Marseille University and Assistance Publique‐Hôpitaux de Marseille Marseille France; ^6^ Clinique des Bronches, Allergies et Sommeil North Hospital, Assistance Publique‐Hôpitaux de Marseille Marseille France

**Keywords:** asthma, bronchial epithelial cells, cell activation, HLA‐F, mobilisation, platelets

## Abstract

Uncontrolled inflammation of the airways in chronic obstructive lung diseases leads to exacerbation, accelerated lung dysfunction and respiratory insufficiency. Among these diseases, asthma affects 358 million people worldwide. Human bronchial epithelium cells (HBEC) express both anti‐inflammatory and activating molecules, and their deregulated expression contribute to immune cell recruitment and activation, especially platelets (PLT) particularly involved in lung tissue inflammation in asthma context. Previous results supported that HLA‐G dysregulation in lung tissue is associated with immune cell activation. We investigated here HLA‐F expression, reported to be mobilised on immune cell surface upon activation and displaying its highest affinity for the KIR3DS1‐activating NK receptor. We explored *HLA‐F* transcriptional expression in HBEC; HLA‐F total expression in PBMC and HBEC collected from healthy individuals at rest and upon chemical activation and HLA‐F membrane expression in PBMC, HBEC and PLT collected from healthy individuals at rest and upon chemical activation. We compared *HLA‐F* transcriptional expression in HBEC from healthy individuals and asthmatic patients and its surface expression in HBEC and PLT from healthy individuals and asthmatic patients. Our results support that HLA‐F is expressed by HBEC and PLT under healthy physiological conditions and is retained in cytoplasm, barely expressed on the surface, as previously reported in immune cells. In both cell types, HLA‐F reaches the surface in the inflammatory asthma context whereas no effect is observed at the transcriptional level. Our study suggests that HLA‐F surface expression is a ubiquitous post‐transcriptional process in activated cells. It may be of therapeutic interest in controlling lung inflammation.

## 1 INTRODUCTION

Uncontrolled airway inflammation is the hallmark of various chronic obstructive lung diseases [asthma, chronic obstructive pulmonary disease (COPD), cystic fibrosis (CF) and non‐CF bronchiectasis]. These diseases are subsequently exacerbated due to acute or chronic infections, accelerated lung function impairment and respiratory insufficiency. These chronic lung diseases and their severe clinical complications constitute a serious public health burden with high morbidity and mortality rates worldwide.^1^

Asthma, which affects 358 million people around the globe and causes 495,000 deaths each year, is characterised by the recruitment, survival and activation of inflammatory cells occurring within the airways, and is associated with structural airway abnormalities.^2^, ^3^

Human bronchial epithelium cells (HBEC) play a main role in immune cells response. Among these, platelets (PLT), anucleate cells implicated in haemostasis and in inflammation,^4^, ^5^, ^6^ appear to have a preferential interaction with lung tissue. In asthma context, HBEC and PLT interaction is supported by PLT activation markers (including PF4 and β‐thromboglobulin) present in broncho‐alveolar lavage (BAL) or the peripheral blood of asthmatic patients.^7^, ^8^, ^9^, ^10^ PLT have also been shown to migrate from blood vessels into lung tissue from asthmatic patients.^10^, ^11^ Specific interaction between lung tissue and PLT is further supported by transfusion related acute lung injury (TRALI), inflammatory adverse events associated with PLT transfusion.^12^

HBEC express immunotolerant and anti‐inflammatory molecules (as the A20 binding protein ABIN‐1, prostaglandin E2 [PGE2] and other lipid mediators as lipoxin A4, resolvins and protectins) that help regulate lung inflammation, and also activating molecules contributing to the recruitment and activation of immune effector cells in response to external harmful agents^13^, ^14^; the activation of pattern recognition receptors (PRRs) expressed by HBEC lead to the release of mediator that attract and activate innate and adaptive immune cells.^13^, ^15^, ^16^, ^17^ HLA class I molecules expression variation is also well described to be associated with activation in most cells including epithelial and immune cells.^18^ However, HLA class I genes display the highest polymorphism of the human genome^19^ and great expression variability is associated with the different HLA allo‐types^18^ or the cell types, especially epithelial cells, immune cells and erythroid and platelet cells.^20^ Similarly, although displaying a restricted genetic polymorphism, HLA‐E expression level is strongly associated with its two main alleles^21^ present at 50% frequencies worldwide.^22^ Therefore, distinguishing expression variation due to cell activation or genetic background is uneasy.

Conversely to these *HLA* genes, HLA‐G expression level has been put forward as a pertinent marker of cell activation and attempt of resolution. HBEC express HLA‐G, which immunotolerant properties have been extensively shown: HLA‐G modulates NK‐ and cytotoxic T‐lymphocyte (CTL)‐mediated activity through interaction with their inhibitory receptors^23^, ^24^, ^25^, ^26^ and its role in lung inflammation control has been supported in lung transplant studies with higher expression in stable recipients than in those with acute rejection or Bronchiolitis Obliterans Syndrome (BOS)^24^ or lower expression associated to de novo Donor Specific Antibody (DSA).^27^ HLA‐G expression is also altered in an asthma context^3^, ^28^; however the modulation of HLA‐G expression may also be driven by genetics: we previously described an association of specific *HLA‐G* haplotypes with severe asthmatic features or poor trend of cystic fibrosis and lung transplantation.^29^, ^30^ HLA‐G expression in healthy bronchial epithelium appears to occur independently of pro‐inflammatory cytokine stimulation (IL‐13 or IL‐5).^25^

The least studied of the HLA‐Ib molecules so far, HLA‐F, is reported to be retained in the cytoplasm and mobilised on the cell surface upon activation of different cell types such as B and T lymphocytes, NK cells, monocytes, and bladder, skin, and liver cell lines.^31^, ^32^

HLA‐F is classified as a HLA‐Ib molecule because of its structure, low genetic diversity and restricted expression pattern. However, HLA‐F has the same structure, but a shorter cytoplasmic tail, compared to HLA‐A, HLA‐B or HLA‐E^33^, ^34^; it displays immature high‐mannose glycans, conversely to HLA‐Ia molecules glycosylation^31^, ^35^; HLA‐F is mainly retain into the cell, notably because of its specific cytoplasmic tail and glycosylation pattern and is expressed at the cell membrane either associated with the β2 microglobulin and peptide or as an open conformer (without β2 micro‐globulin and peptide)^31^, ^33^, ^36^; peptides presented by HLA‐F, from nucleus or cytosol, reach seven to over 30 amino‐acids.^37^, ^38^ Moreover, contrary to HLA‐G and HLA‐E which display the highest affinity for inhibitory immune receptors, HLA‐F has its highest affinity for the KIR3DS1‐activating NK receptor^33^, ^39^ and the binding thereof can trigger NK cytotoxicity and IFN‐g production. KIR3DS1 has been linked to the outcome of HIV and other viral infections, cancer immune monitoring, autoimmune disease and transplant outcome.^33^, ^40^, ^41^

Sparse data are available regarding HLA‐F expression upon cell activation in cells other than lymphocytes or carcinogenic cells.^31^, ^32^, ^36^

Because of its implication in immune response in inflammatory context, deciphering HLA‐F surface expression in cells involved in asthma pathogenesis, as HBEC and PLT, would contribute to a better understanding of inflammation onset and immune response in chronic lung disease. The interaction between HLA‐F with its receptors expressed by immune effector cells may also be of therapeutic interest in controlling inflammation as it was successfully shown for HLA‐E and its receptors.^42^

In this study, we explored *HLA‐F* transcriptional expression in HBEC from healthy individuals and we analysed HLA‐F membrane expression in PBMC, HBEC and PLT collected from healthy individuals at rest and upon chemical activation. We then compared *HLA‐F* transcriptional expression in HBEC and its membrane‐bound expression in HBEC and PLT from healthy individuals and asthmatic patients.

## 2 MATERIAL AND METHODS

### 2.1 Primary cells

Peripheral Blood Mononuclear Cells (PBMC) were obtained from healthy donors (healthy individuals [HI], *N* = 10). A medical interview was conducted prior to blood donation to exclude donors with medical contraindications such as severe asthma or respiratory failure. The donations were collected in accordance with French blood donation regulations and ethics, and the French Public Health Code (Article L.1221‐1). PBMC cells were isolated from EDTA peripheral blood collected 24 h before processing by density gradient centrifugation (Lymphoprep solution, StemCell) and cultured in FBS (Fetal Bovine Serum) supplemented RPMI overnight.

Human bronchial epithelial cells (HBEC) were obtained from healthy donors (human transplant donor lungs that did not match criteria for transplantation^43^ and donated to medical research) (healthy individuals [HI], *N* = 6) and from asthmatic patients (lung biopsy) (severe asthmatic patients [SA], *N* = 6).

Primary human bronchial epithelial cells were isolated by protease digestion of human airways, and cells were cultivated under Air‐Liquid Interface (ALI) conditions, as previously described.^15^ HBEC were maintained in culture for 21 days to obtain a differentiated cell population with a mucociliary phenotype. This model of ex vivo differentiated epithelia was previously assessed by epithelial and sub epithelial layers, mucus production, IL‐8 release, lipoxin A generation, mucin production, and lipoxygenase gene expression.^15^ A differentiated cell population with mucociliary phenotype displaying asthmatic characteristics was obtained from the primary HBEC of patients presenting severe asthma.

RNA previously obtained from HBEC from healthy individuals and asthmatic patients (*N* = 12 and *N* = 12 respectively)^28^ and cultured in ALI conditions was included for transcriptional analysis.

Platelets (PLT) were obtained from healthy donors and asthmatic patients (HI, *N* = 6 and SA, *N* = 6). PLT were purified according to standard procedures. Briefly, blood was collected in tubes containing 3.8% sodium citrate (0.129 M) and centrifuged at 180 g for 10 min to obtain platelet‐rich plasma (PRP). PLT were pelleted by centrifugation of PRP supplemented with apyrase (1 unit/ml) at 1000 g for 5 min. PLT were then washed using Tyrode's buffer (138 mM NaCl, 2.7 mM KCl, 12 mM NaHCO_3_, 0.4 mM NaH_2_PO_4_, 1 mM MgCl_2_, 2 mM CaCl_2_, 5 mM Hepes, 3.6 mg/ml BSA and 5.5 mM glucose, pH 7.3) supplemented with 0.02 unit/ml apyrase, 10 units/ml heparin and 175 ng/mL prostaglandin E1. The final PLT pellet was suspended in PneumaCult™‐ALI Medium (Stemcell) at a final concertation of 10^8^ PLT/ml and immediately processed.

Asthma was classified as severe by using the current Global Initiative for Asthma guidelines (www.ginasthma.org). Patients with severe asthma met the American Thoracic Society criteria for refractory asthma.^44^ The ethics committees of the institutions involved approved this study (CERC‐SFCTCV‐2018‐5‐6‐9‐8‐32‐DjXa and COBRA, Cohorte Obstruction Bronchique et Asthme—Bronchial Obstruction and Asthma Cohort, INSERM Hôpital Bichat, CNIL 28/01/2008). Subjects' characteristics are presented in Table 1.

**TABLE 1:** Subject characteristics

Subjects	*N*	Female (%) (M/F)	Age	FEV1 (%)	ICS (%)	LABA (%)	OCS (%)	Asthma control (%)	Exacerbation in the past year (%)
HI (PBMC)	10	70.0 (3/7)	49 [21–60]	NA	0	0	0	NA	NA
HI (Cells)	6	83.3 (1/5)	48 [23–64]	NA	0	0	0	NA	NA
SA (Cells)	6	33.3 (4/2)	49 [36–67]	80 [36–112]	100	100	30	50	50
HI (RNA)	12	33.3 (8/4)	46 [17–61]	NA	0	0	0	NA	NA
SA (RNA)	12	25 (9/3)	59 [47–71]	70 [60.4–85]	100	100	57	42	58

*Note*: Healthy individuals (HI), severe asthmatic patients (SA), number of subject (*N*), female percentage (%), male/female (M/F), age (median and min–max), FEV1 (forced expiratory volume in 1 s), ICS (inhaled corticosteroid), LABA (long acting β2 agonist), OCS (oral corticosteroid), asthma control, and exacerbation during the past year are described.

Abbreviation: NA, not applicable.

### 2.2 HLA‐F expression in PBMC, HBEC and PLT from healthy individuals

#### 2.2.1 HLA‐F transcriptional expression in HBEC from healthy individuals

*HLA‐F* transcriptional expression was investigated by real‐time PCR in RNA extracted from HBEC from healthy individuals (*N* = 12).

Total RNA, isolated using the RNeasy kit (Qiagen, France), was reverse‐transcribed using Superscript IV Reverse Transcriptase (Invitrogen) and real‐time PCR analyses were performed using TaqMan technology (Life Technologies). *HLA‐F* transcriptional expression was quantified using TaqMan assay (Hs04193807_g1, Invitrogen) and *ACTB* (actinβ) was used as an endogenous control (*ACTB* Hs99999903_m1, Invitrogen). Each experiment was carried out in duplicate using a QwantStudio 3 (Invitrogen).

The average Ct was calculated with StepOne 2.1 software (Invitrogen), excluding Ct duplicates with a standard deviation above 0.5.

#### 2.2.2 Chemical activation of PBMC, HBEC and PLT from healthy individuals

PBMC, HBEC and PLT from healthy individuals were analysed at resting state and following phorbol 12‐myristate 13‐acetate (PMA)/ionomycin (PI) activation in order to confirm that HLA‐F is expressed and is retained in the cytoplasm under healthy physiological conditions, as previously described in other cell types.^31^, ^32^

PMA (Abcam) and ionomycin (Sigma‐Aldrich) were solubilised in DMSO and used at a final concentration of 0.2 and 20 μg/ml, respectively.^45^ Dimethyl sulfoxide (DMSO) and unstimulated cells (UNS) were used as reference and negative controls, respectively. PI activation was performed for 24 h (PBMC), 48 h (HBEC) or overnight (PLT) at 37°C according to experimental optimization based on cell viability vs. HLA‐F surface expression (data not shown).

Incubation with thrombin receptor activator peptide 6 (Trap 6) (Sigma‐Aldrich) was used as the PLT activation control (20 μM for 10 min).^46^

PI efficient activation was assessed by cytometry analysis using activating markers: the CD25 (alpha chain of the IL2 receptor) for PBMC and the P‐selectin expression for the PLT; respectively using the murine IgG2 anti‐CD25 antibody clone T07774‐PE (Beckman Counter) and the anti‐CD62P recombinant antibody clone REA389‐PE (Miltenyi) with the recombinant human IgG1 as isotype (clone REA293‐PE, Miltenyi).

#### 2.2.3 HLA‐F total expression in PBMC and HBEC from healthy individuals according to chemical activation

The HLA‐F protein content analysis was carried out by Western blot in PBMC (N = 5) and in HBEC from healthy individuals (*N* = 3). HLA‐F differential expression level according to chemical activation (unstimulated, DMSO treatment and PI treatment) was also explored.

Western blot analyses were performed as previously described^28^ with HLA‐F polyclonal antibody clone PA5100066 (Invitrogen). Load normalisation was carried out with anti‐actinβ and the densitometric analysis of immunoblots was performed by using Alphaview Software (ProteinSimple).

#### 2.2.4 HLA‐F membrane‐bound expression in PBMC, HBEC and PLT from healthy individuals according to chemical activation

Cytometry analysis was used to explore HLA‐F membrane‐bound expression in PBMC (*N* = 10), HBEC (*N* = 6) and PLT (*N* = 6) from healthy individuals and to explore HLA‐F mobilisation at cell surface upon chemical activation (unstimulated, DMSO treatment and PI treatment).

HLA‐F expression was investigated using the murine IgG1 antibody clone 3D11‐PE (Biologends) that detects all forms of HLA‐F.^47^, ^48^ The murine IgG1 antibody clone 679.1Mc7‐PE (Beckman Coulter) served as the isotype control.

HBEC viability was assessed by SYTOX Blue Dead Cell Staining (S34857, Invitrogen). PLT purity was assessed by PLT‐specific anti‐CD41a murine IgG1 antibody staining (clone REA386‐APCVio®770; Miltenyi Biotec)^49^; the murine IgG1 antibody clone REA293‐APCVio®770 (Miltenyi Biotec) served as the isotype control.

PBMC and HBEC cytometry data were acquired on a Cytoflex machine (Beckman Coulter) and analysed with CytExpert 2.3 software (Beckman Coulter). As the Cytoflex machine vortexes tube before acquisition and because of the fragile nature of PLT, PLT cytometry data were acquired on a MACSQuant machine (Miltenyi Biotec) and analysed with FlowJo 10 software (BDbiosciences).

The cell population was detected according to its morphology using an initial gate set in a FSC‐A/SSC‐A plot. Cells clump and doublets were excluded by gating single cells in FSC‐A/FSC‐H plot. Median of MFI (Mean Fluorescence Intensity) values and percentage of cells expressing HLA‐F were gated in the subsequent PE‐A/SSC‐A plot. Illustration of cytometric detection of PBMC, HBEC and PLT populations, HBEC cell viability, PLT purity and HLA‐F membrane expression are respectively shown in Figures [Link], [Link].

### 2.3 HLA‐F transcriptional expression and membrane‐bound expression in HBEC and PLT according to asthmatic status

*HLA‐F* transcriptional expression, investigated by Q‐PCR as described above, was compared between RNA extracted from HBEC from healthy individuals (N = 12) and from severe asthmatic patients (*N* = 12).

HLA‐F membrane‐bound expression was investigated by cytometry as described above in HBEC (*N* = 6) and PLT (*N* = 6) from asthmatic patients and compared to healthy individuals ‐ without chemical activation (both cell types, *N* = 6).

### 2.4 Statistical analyses

Q‐PCR Ct mean data are shown and results are expressed as dCt (delta of cycle threshold, expression normalised by *ACTB* endogenous gene) with median and range [min, max].

Results of HLA‐F total expression explored by Western blot are expressed as the ratio of HLA‐F/actinβ bands mean intensity (A.U.: arbitrary unit).

HLA‐F membrane‐bound expression explored by cytometry was estimated by median MFI and as a percentage of expressing cells.

HLA‐F total expression and mobilisation at cell membrane were estimated at resting state (no activation) and compared to PI activation and incubation with the PI solvent (DMSO); folds of induction between the experimental conditions are shown.

HLA‐F transcriptional expression and expression at membrane cells according to asthma status was investigated by comparison of asthmatic patients with healthy individuals without chemical activation; folds of induction between the clinical conditions are shown.

All association and correlation tests were performed with GRAPH PAD Prism 9 software. The differences between two modalities were tested using an unpaired t‐test. Ordinary one‐way ANOVA followed by Holm‐Sidak multiple comparisons test were used to test more than two modalities.

## 3 RESULTS

### 3.1 HLA‐F is expressed and retained in the cytoplasm in PBMC, HBEC and PLT from healthy individuals

**FIGURE 1:**
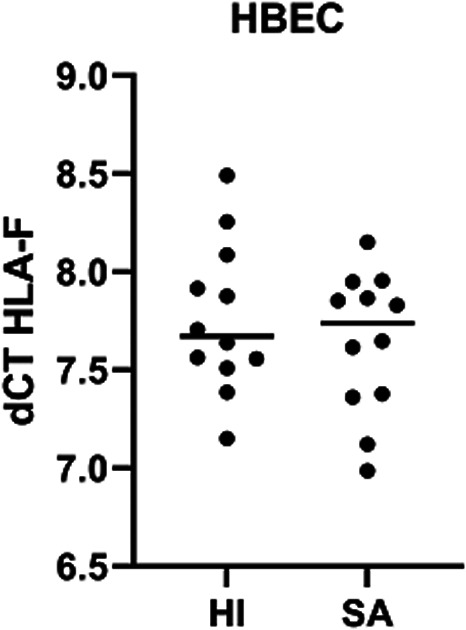
HLA‐F transcriptional expression in HBEC according to asthmatic status (dCt: delta of cycle threshold, expression quantified by Q‐PCR normalised by ACTB endogenous gene) (HBEC HI, *N* = 12, mean dCT = 7.762 [7.151–8.492]; (HBEC SA, *N* = 12, mean dCT = 7.643 [6.986–8.152]; *t* test *p* = 0.44)

HBEC from healthy individuals displayed *HLA‐F* transcriptional expression (Figure 1, mean dCT of 7.76 [7.15–8.49]; Ct = 16.78 [16.14–17.52] and 24.54 [23.52–25.29] for *ACTB* and *HLA‐F*, respectively).

Western blot data confirm that PBMC from healthy individuals express HLA‐F and show that HBEC from healthy individual express HLA‐F protein (Table 2 and Figure S5).

**TABLE 2:** HLA‐F protein total expression explored by Western blot in PBMC and HBEC protein lysates from healthy individuals (HI) according to stimulation conditions (unstimulated (UNS); DMSO; PMA/Ionomycin (PI))

Cell	*N*	Stimulation	HLA‐F	actinβ	Ratio	Folds of induction
PBMC	5	UNS	4802 [776.5–10,413]	18,652 [13544–22,702]	0.25 [0.04–0.50]	1.0
		DMSO	6902 [1662–12,809]	21,800 [12326–28,339]	0.30 [0.13–0.51]	1.2
		PI	1591 [577.9–2945]	8389 [4202–11,556]	0.27 [0.05–0.70]	1.1
HBEC	3	UNS	9060 [1269–16,554]	7621 [6924–8435]	1.16 [0.17–1.96]	1.0
		DMSO	8058 [244.0–15,005]	8578 [7092–10,779]	0.99 [0.03–2.12]	0.9
		PI	9037 [3070–17,027]	8983 [5991–11,928]	0.90 [0.51–1.43]	0.8

*Note*: HLA‐F and actinβ protein expression are shown as bands mean intensity (A.U.: arbitrary unit) and ratio. Folds of induction between the mean ratios of experimental conditions as compared to unstimulated (UNS) condition are shown.

Cytometry data confirm that PBMC from healthy individuals display low levels of HLA‐F expression on the cell surface and show similarly low expression at cell surface in HBEC and PLT from healthy individuals (Table 3). Expression intensities (MFI mean) were three times higher in HBEC than in PBMC (*p* < 0.001), although percentage of expressing cells were similar (*p* = 0.957). No comparison was attempted with PLT as analyses were performed with another cytometer, however, percentage of expressing cells appeared three times lower.

**TABLE 3:** HLA‐F membrane expression explored by cytometry in PBMC, HBEC and PLT from healthy individuals (HI) according to stimulation conditions (unstimulated [UNS]; DMSO; PMA/ionomycin [PI]) and from asthmatic patients (AS)

Cell	*N*	Donor	Stimulation	MFI	Folds of induction (MFI)	Percentage	Folds of induction (percentage)
PBMC	10	HI	UNS	266.1 [111.2–416.5]	1.0	8.3 [3.0–14.2]	1.0
			DMSO	101.9 [40.4–191.0]	0.4	2.2 [0.3–3.8]	0.3
			PI	1248 [527.4–1651]	4.7	68.3 [28.5–82.0]	8.2
HBEC	6	HI	UNS	822.6 [673.0–2244]	1.0	7.5 [4.1–29.2]	1.0
			DMSO	1595 [522.9–2269]	1.9	7.2 [3.0–16.2]	1.0
			PI	2769 [834.0–5421]	3.4	45.6 [16.4–75.4]	6.1
	6	SA	UNS	2915 [1905–4395]	11.0	31.9 [9.1–63.6]	3.8
PLT	6	HI	UNS	0.6 [0.5–0.7]	1.0	2.5 [1.4–4.0]	1.0
			DMSO	0.6 [0.5–0.8]	1.0	2.5 [1.0–4.4]	1.0
			PI	0.9 [0.8–0.9]	1.5	7.0 [5.6–11.9]	2.8
	6	SA	UNS	0.9 [0.7–1.1]	1.5	8.2 [3.1–15.5]	3.3

*Note*: Number (*N*) of patients, MFI median expression (MFI, median and range) and percentage of cell expressing HLA‐F (percentage, median, and range) are shown. Folds of induction between experimental conditions in cells from healthy individuals (HI) as compared to unstimulated (UNS) condition; and between asthmatic patients (AS) as compared to HI in unstimulated (UNS) condition are shown.

PMA/ionomycin (PI) activation was assessed using standard markers: PI activation of PBMC significantly increased CD25 (alpha chain of the IL2 receptor) staining, and PI and Trap6 used separately to activate PLT significantly increased P‐selectin staining (data not shown).

In PBMC and HBEC, total HLA‐F protein expression, analysed by Western blot, was not influenced by PI activation (Table 2, *p* = 0.927 and *p* = 0.933 for PBMC and HBEC respectively), whereas cytometry analyses support that PI activation significantly mobilised HLA‐F on the cell surface for the three cell types compared to unstimulated cells (UNS) or cells incubated with DMSO. As compared to unstimulated cells, expression of HLA‐F at cell surface upon PI treatment was 8.2 and 6.1 times higher for PBMC and HBEC respectively (Table 3 and Figures [Link], [Link]). Noteworthy, as comparted to PBMC and HBEC, folds of induction appeared lower for PLT as PLT treated with PI express 2.8 times more HLA‐F at cell surface than unstimulated ones.

Our data support the fact that HLA‐F is expressed in PBMC, HBEC and PLT from healthy individuals but is retained in the cytoplasm at resting state.

### 3.2 HBEC and PLT from severe asthmatic patients express higher membrane‐bound HLA‐F levels independently of transcriptional level

Transcriptional *HLA‐F* expression in HBEC from healthy individuals and asthmatic patients showed no difference (mean *HLA‐F* Ct = 24.54 [23.52–25.29] and 24.07 [23.30–24.52] respectively; Figure 1), supporting that the asthma context did not impact *HLA‐F* transcriptional expression level.

Analysis of HLA‐F membrane‐bound expression on HBEC and PLT obtained from severe asthmatic patients revealed significantly higher levels compared to healthy individuals. Both cells types displayed similar folds of induction, with respective values of 3.8 and 3.3 (Table 3 and Figures [Link], [Link]). As membrane‐bound HLA‐F is expressed at low level at resting state, especially at PLT surface, folds of induction must be considered cautiously.

Our data support that airway inflammation in an asthmatic context impacts HLA‐F mobilisation on HBEC and PLT surfaces independently of transcriptional level.

## 4 DISCUSSION

Severe asthma, one of the most common inflammatory lung diseases, is characterised by uncontrolled recruitment and activation of inflammatory cells within the airways.^2^, ^3^ The inflammation of bronchial epithelium cells and their interaction with immune cells, particularly PLT, are well documented in asthma^5^, ^6^, ^7^, ^10^, ^11^, ^13^, ^15^, ^16^, ^17^; and PLT activation has been demonstrated by PLT mediators release in BAL and peripheral blood from patients with severe asthma.^8^, ^9^, ^10^, ^11^

We investigated an airway inflammation marker expressed both by bronchial epithelium cells and by peripheral circulating PLT in an inflammatory context such as asthma, to get a better insight of inflammation onset and possible epithelial‐immune cells positive loop in chronic lung disease.

We previously showed that HBEC express HLA‐G according to genetics and asthma status.^28^ In this study, we focused on HLA‐F, another HLA‐Ib molecule, known for its membrane expression upon cell activation and involvement in immune response.

Our study shows that *HLA‐F* is transcribed into HBEC. These results are consistent with transcriptome and RNAseq analyses (BioProjects: PRJEB2445; PRJEB4337 and PRJNA280600) demonstrating that *HLA‐F* is highly transcribed in lung and white blood cells. Our results confirm that HLA‐F is expressed at low levels on the PBMC surface,^31^ and support its low expression level on the surface of HBEC and PLT obtained from healthy individuals. HLA‐F is mobilised on healthy individual HBEC and PLT surfaces upon chemical activation, as previously shown in PBMC.^32^ These results support the fact that *HLA‐F* is transcribed and translated in HBEC and PLT but is retained in the cytoplasm with low surface expression at baseline physiological conditions. Mechanistic studies supported that HLA‐F is retained into the cell because of its peculiar glycosylation pattern (immature high‐mannose glycans) as compared to other HLA‐Ia.^31^, ^35^

We then compared *HLA‐F* transcriptional level in HBEC and HLA‐F membrane‐bound expression in HBEC and PLT from asthmatic patients and healthy individuals. Our results corroborate the fact that HLA‐F surface expression on both HBEC and PLT surface is linked to asthmatic status whereas no variation is observed at the transcriptional level in HBEC. The mobilisation of HLA‐F at PLT surface seems less pronounced than in HBEC from asthmatic patients.

The implication of HLA‐F mobilisation on the cell surface in the inflammatory asthma context needs to be addressed as immune effector cell activity is determined by the balance of inhibitory and stimulatory signals received. HLA‐F displays its highest affinity for the KIR3DS1‐activating NK receptor and triggers NK cytotoxicity and IFN‐g production.^33^, ^39^ *KIR3DS1* is a common allelic variant of the *KIR3DL1* gene with an allelic frequency that varies greatly in the population (from 0% to 100%) and encodes an activating receptor which unique ligand is HLA‐F, whereas KIR3DL1 is an inhibitory KIR receptor that binds to Bw4 allotypes.^50^, ^51^ KIR3DS1 has been associated with the outcome of HIV and other viral infections, cancer immune monitoring, autoimmune disease and transplant outcome.^33^, ^39^ HLA‐F also binds to inhibitory receptors,^52^ as HLA‐G and HLA‐E.

HLA‐F membrane‐bound expression was also described on placental extravillous trophoblasts (reviewed in^53^) and has recently been linked to cell proliferation in glioma.^54^ Thorough investigation is warranted to specify the role of this molecule in structural changes observed in asthmatic airways affecting the bronchial epithelium.

Our study supported that HLA‐F is expressed at cell surface in asthmatic context in both PLT and HBEC cells, however we did not explore mechanistic insights concerning HLA‐F mobilisation at cell surface or HLA‐F conformation expression; studies are also needed to investigate the association of HLA‐F expression according to the magnitude of inflammation i.e. asthma severity and other lung disease (COPD, CF and non‐CF bronchiectasis). Those results may be of interest to validate a surrogate marker of lung inflammation using a method avoiding lung biopsy.

## AUTHOR CONTRIBUTIONS

Julien Paganini, Christophe Picard, Jacques Chiaroni, Pascal Chanez, Delphine Gras and Julie Di Cristofaro contributed to the study concept and design. Sabrina Fiouane and Julie Di Cristofaro organised the database. Sabrina Fiouane, Mohamad Chebbo and Julie Di Cristofaro performed the statistical analysis. Julie Di Cristofaro wrote the first draft of the manuscript. Sabrina Fiouane, Mohamad Chebbo and Pascal Chanez wrote sections of the manuscript. All of the authors contributed to the manuscript review and have read and approved the final version.

## FUNDING INFORMATION

This work was supported by Fondation du Souffle, Fonds de Dotation Recherche en Santé Respiratoire (2017).

## CONFLICT OF INTEREST

The authors declare that the research was conducted in the absence of any commercial or financial relationships that could be construed as a potential conflict of interest.

## Supporting information


**Figure S1** One exemplary of PBMC (1A), HBEC (1B) and PLT (1D) population analysis by cytometry according to their morphology using an initial gate set in a FSC‐A/SSC‐A plot. One exemplary of single cell (HBEC) gated in a FSC‐A/FSC‐H plot is shown (1C).

**Figure S2** One exemplary of HBEC cell viability (HBEC stain with SYTOX Blue Dead Cell is shown in light grey vs. unstained HBEC in grey), percentage of dead cells are indicated.

**Figure S3** One exemplary of PLT purity assessed by PLT‐specific staining. Isotype staining is shown in light grey and anti‐CD41a staining in grey.

**Figure S4** HLA‐F expression in PBMC (4A), HBEC (4B) and PLT (4C) from healthy individuals (PE‐A/SSC‐A Histogram). Isotype staining is shown in light grey, 3D11 staining with no activation in grey and 3D11 staining following PI activation in dark grey.

**Figure S5** Representative western blot analysis of PBMC and HBEC cells according to stimulation conditions (unstimulated (UNS); DMSO; PMA/Ionomycin (PI)). Total protein levels of HLA‐F were monitored; actinβ staining was employed to normalise loading.

**Figure S6** HLA‐F membrane expression in PBMC from healthy individuals (HI, N = 10) according to stimulation conditions (unstimulated (UNS); DMSO; PMA/Ionomycin (PI)). MFI median expression (6A) and the percentage of cells expressing HLA‐F (6B) displayed a statistically significant difference; Ordinary one‐way ANOVA p < 0.001, respectively.

**Figure S7** HLA‐F membrane expression in HBEC from healthy individuals (HI, N = 6) according to stimulation conditions (unstimulated (UNS); DMSO; PMA/Ionomycin (PI)) MFI median expression (7A) and percentage of cell expressing HLA‐F (7B) displayed a statistically significant difference; Ordinary one‐way ANOVA p = 0.048 and p = 0.001, respectively.

**Figure S8** HLA‐F membrane expression in PLT from healthy individuals (HI, N = 6) according to stimulation conditions (unstimulated (UNS); DMSO; PMA/Ionomycin (PI)). MFI median expression (8A) and percentage of cells expressing HLA‐F (8B) displayed a statistically significant difference; Ordinary one‐way ANOVA p < 0.001, respectively.

**Figure S9** HLA‐F membrane expression in HBEC according to asthmatic status (healthy individuals (HI, N = 6) and severe asthmatic patients (SA, N = 6)) (MFI median expression (9A) and percentage of cells expressing HLA‐F (9B) displayed a statistically significant difference; t‐test p = 0.003 and p = 0.017 respectively).

**Figure S10** HLA‐F membrane expression in PLT according to asthmatic status (healthy individuals (HI, N = 6) and severe asthmatic patients (SA, N = 6)) (MFI median expression (10A) and percentage of cells expressing HLA‐F (10B) displayed a statistically significant difference; t‐test p = 0.004 and p = 0.007, respectively).

## Data Availability

The data that support the findings of this study are available from the corresponding author upon reasonable request.
