# Supplementary figures and images for: Mobilisation of HLA‐F on the surface of bronchial epithelial cells and platelets in asthmatic patients
Source: HLA. 2022 Aug 30;100(5):491–9. doi: 10.1111/tan.14782 (PMC9804204; doi:10.1111/tan.14782)

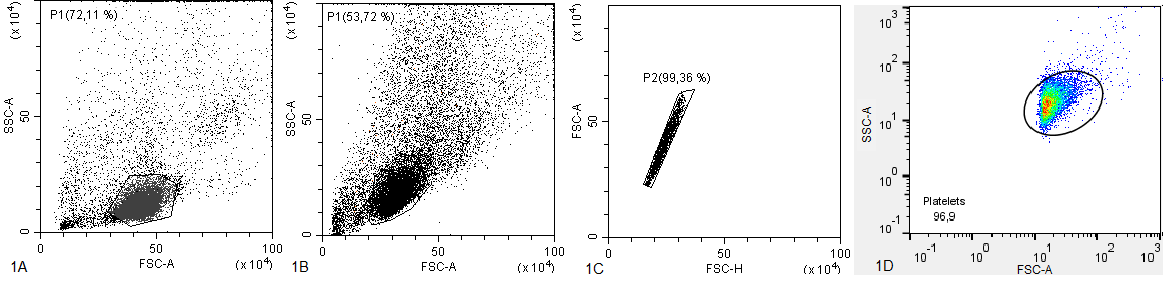

Supplement: Supplementary file 1 — Figure S1 One exemplary of PBMC (1A), HBEC (1B) and PLT (1D) population analysis by cytometry according to their morphology using an initial gate set in a FSC‐A/SSC‐A plot. One exemplary of single cell (HBEC) gated in a FSC‐A/FSC‐H plot is shown (1C). [file TAN-100-491-s006.tif]

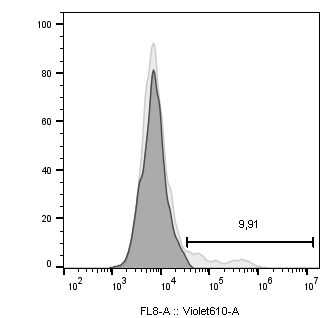

Supplement: Supplementary file 2 — Figure S2 One exemplary of HBEC cell viability (HBEC stain with SYTOX Blue Dead Cell is shown in light grey vs. unstained HBEC in grey), percentage of dead cells are indicated. [file TAN-100-491-s007.tif]

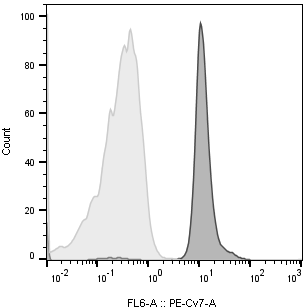

Supplement: Supplementary file 3 — Figure S3 One exemplary of PLT purity assessed by PLT‐specific staining. Isotype staining is shown in light grey and anti‐CD41a staining in grey. [file TAN-100-491-s005.tif]

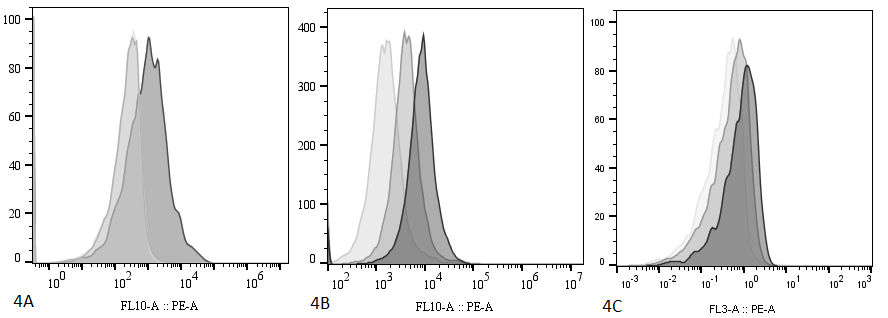

Supplement: Supplementary file 4 — Figure S4 HLA‐F expression in PBMC (4A), HBEC (4B) and PLT (4C) from healthy individuals (PE‐A/SSC‐A Histogram). Isotype staining is shown in light grey, 3D11 staining with no activation in grey and 3D11 staining following PI activation in dark grey. [file TAN-100-491-s010.tif]

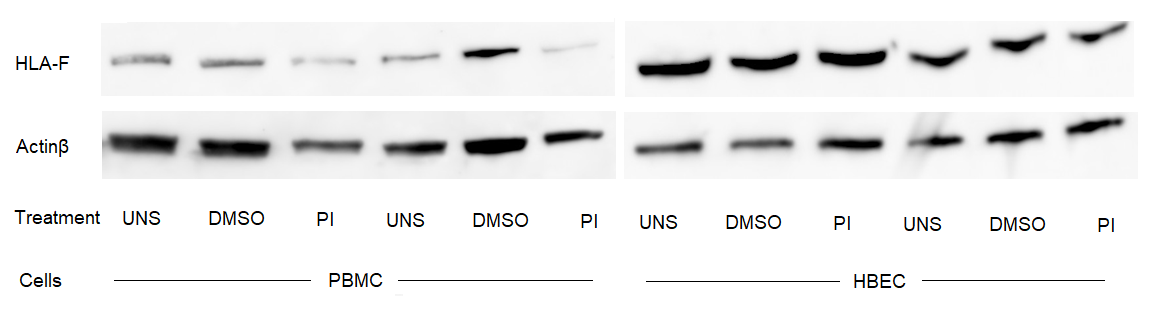

Supplement: Supplementary file 5 — Figure S5 Representative western blot analysis of PBMC and HBEC cells according to stimulation conditions (unstimulated (UNS); DMSO; PMA/Ionomycin (PI)). Total protein levels of HLA‐F were monitored; actinβ staining was employed to normalise loading. [file TAN-100-491-s001.png]

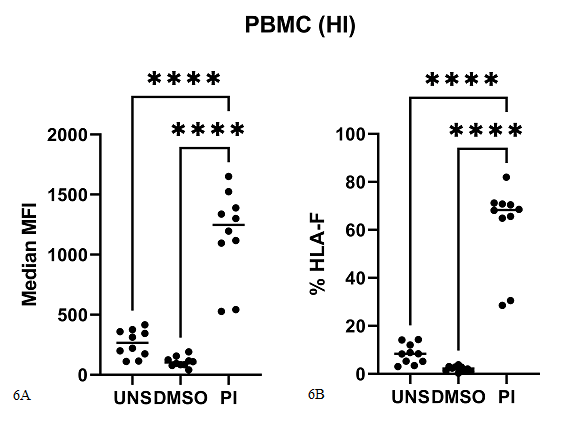

Supplement: Supplementary file 6 — Figure S6 HLA‐F membrane expression in PBMC from healthy individuals (HI, N = 10) according to stimulation conditions (unstimulated (UNS); DMSO; PMA/Ionomycin (PI)). MFI median expression (6A) and the percentage of cells expressing HLA‐F (6B) displayed a statistically significant difference; Ordinary one‐way ANOVA p < 0.001, respectively. [file TAN-100-491-s009.png]

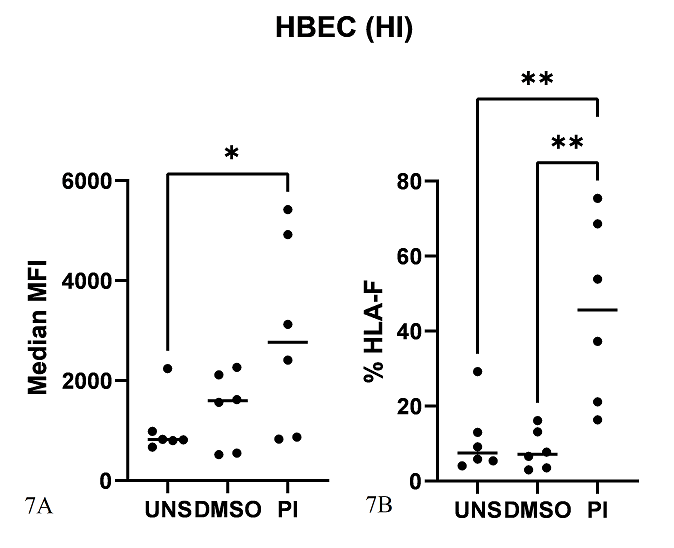

Supplement: Supplementary file 7 — Figure S7 HLA‐F membrane expression in HBEC from healthy individuals (HI, N = 6) according to stimulation conditions (unstimulated (UNS); DMSO; PMA/Ionomycin (PI)) MFI median expression (7A) and percentage of cell expressing HLA‐F (7B) displayed a statistically significant difference; Ordinary one‐way ANOVA p = 0.048 and p = 0.001, respectively. [file TAN-100-491-s003.png]

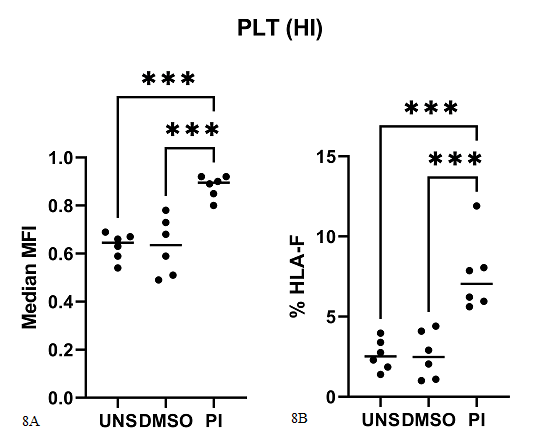

Supplement: Supplementary file 8 — Figure S8 HLA‐F membrane expression in PLT from healthy individuals (HI, N = 6) according to stimulation conditions (unstimulated (UNS); DMSO; PMA/Ionomycin (PI)). MFI median expression (8A) and percentage of cells expressing HLA‐F (8B) displayed a statistically significant difference; Ordinary one‐way ANOVA p < 0.001, respectively. [file TAN-100-491-s004.png]

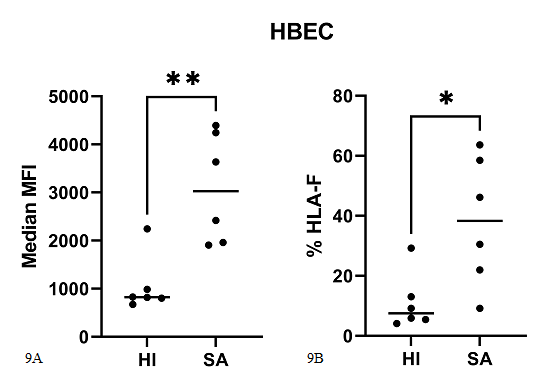

Supplement: Supplementary file 9 — Figure S9 HLA‐F membrane expression in HBEC according to asthmatic status (healthy individuals (HI, N = 6) and severe asthmatic patients (SA, N = 6)) (MFI median expression (9A) and percentage of cells expressing HLA‐F (9B) displayed a statistically significant difference; t‐test p = 0.003 and p = 0.017 respectively). [file TAN-100-491-s008.png]

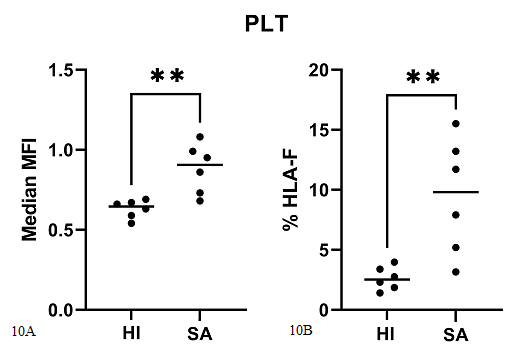

Supplement: Supplementary file 10 — Figure S10 HLA‐F membrane expression in PLT according to asthmatic status (healthy individuals (HI, N = 6) and severe asthmatic patients (SA, N = 6)) (MFI median expression (10A) and percentage of cells expressing HLA‐F (10B) displayed a statistically significant difference; t‐test p = 0.004 and p = 0.007, respectively). [file TAN-100-491-s002.png]
